# Resilience of the gelatinous zooplankton species *Oikopleura dioica* to ocean alkalinity enhancement

**DOI:** 10.1371/journal.pone.0344503

**Published:** 2026-03-30

**Authors:** Amrita Bhaumik, Nicolás Sánchez, Silvan Urs Goldenberg, Synne Spjelkavik, María Couret, Ulf Riebesell, Maarten Boersma, Cornelia Jaspers

**Affiliations:** 1 Alfred-Wegener-Institut Helmholtz-Zentrum für Polar- und Meeresforschung, Biologische Anstalt Helgoland, Helgoland, Germany; 2 Department of Coastal Systems, Royal Netherlands Institute for Sea Research (NIOZ), Den Burg, the Netherlands, GEOMAR Helmholtz Centre for Ocean Research Kiel, Kiel, Germany; 3 Centre for Gelatinous Plankton Ecology and Evolution, DTU Aqua - Technical University of Denmark, Lyngby, Denmark; 4 Instituto de Oceanografíay Cambio Global (IOCAG), Universidad de Las Palmas de Gran Canaria, Unidad Asociada, Universidad de Las Palmas de Gran Canaria-Consejo Superior de Investigaciones Científicas (ULPGC-CSIC), Telde, Spain; 5 Fachbereich 2 Biologie/Chemie, Universität Bremen, Bremen, Germany; 6 Alfred-Wegener-Institut Helmholtz-Zentrum für Polar- und Meeresforschung, Wadden Sea Station, Sylt, Germany; VIT University, INDIA

## Abstract

Ocean alkalinity enhancement (OAE) through mineral dissolution is a promising marine carbon dioxide removal strategy because it increases the buffering capacity of seawater and thereby enhances passive storage of atmospheric CO_2_. However, the ecological consequences of OAE for zooplankton, particularly gelatinous species, remain poorly understood. Here, we assessed the response of a key gelatinous zooplankton species to OAE in a 53-day mesocosm experiment in a temperate Norwegian fjord*. Oikopleura dioica* is a globally distributed zooplankton member, known for its high secondary production capacity and key role in vertical carbon flux. *O. dioica* continuously produces mucous feeding structures (‘houses’), which efficiently retain submicron particles. Once discarded, these houses can sink rapidly and contribute to vertical carbon exports. To test the impacts of OAE on *O. dioica* abundances and their house production capacity, we exposed natural plankton communities to non-CO_2_-equilibrated OAE scenarios spanning a ΔTA range from 0–600 μmol kg^-1^, using silicate-based (olivine) and calcium-based (slaked lime) minerals. Population dynamics of *O. dioica* were monitored alongside the plankton community, and targeted bottle incubations were used to quantify house production and feeding rates. We show that *O. dioica* abundances varied by an order of magnitude within and across treatments. No interaction between *O. dioica* abundance and alkalinity levels or mineral types could be detected. Instead, *O. dioica* abundance variations were primarily explained by prey availability (picoplankton). Additionally, house production and feeding rate experiments showed that *O. dioica* were unaffected by OAE across all treatments. These findings indicate that *O. dioica,* as a key gelatinous zooplankton member, is physiologically resilient to OAE within the tested range. Future studies should incorporate gelatinous zooplankton into OAE assessments and investigate higher alkalinity perturbations to evaluate potential ecosystem impacts and larvacean-mediated changes in carbon export during under OAE deployments above ΔTA 600 μmol kg^-1^.

## Introduction

Since the Industrial Revolution, anthropogenic carbon dioxide (CO_2_) emissions have risen at an unprecedented rate. So far, the oceans have been absorbing about 30% of these emissions [1]. While this uptake has facilitated the modulation of global warming, it has come at a cost. The additional CO_2_ has altered the ocean’s carbonate chemistry, leading to a decline in pH, a process known as ocean acidification, with wide-ranging impacts on marine life [2,3]. Since pre-industrial times, global mean surface ocean pH has decreased by about 0.1 units (corresponding to a **~** 30% increase in hydrogen ion concentration) and is projected to decline by another 0.1–0.3 units under intermediate to high-emission scenarios until 2100 [4]. To counteract ocean acidification, restore the ocean’s natural buffering system, and increase the CO_2_ uptake capacity, ocean alkalinity enhancement (OAE) has gained attention as a marine carbon dioxide removal (mCDR) and ocean restoration strategy [5–7]. Recent assessments, including IPCC AR6, now explicitly recognise OAE as an ocean-based CDR strategy that can contribute to meeting climate targets [8]. However, it is also emphasised to carefully evaluate associated ecological risks along with potential benefits [8]. In parallel, dedicated international and national research initiatives such as OceanNETs (EU Horizon2020 project) or CDRmare (German Allianz Ocean-research – DAM) have been working on generating data to determine monitoring requirements and guidelines for a ‘safe-operating-space’ [9–11].

Mechanistically, OAE accelerates the natural weathering of rock, whereby alkaline ions are released and increase the total alkalinity (TA) of seawater, which stabilizes the pH and restores the natural buffer capacity [7,12–14]. This process has been documented as effective in regulating the climate over geological timescales [15]. However, naturally occurring OAE through rock weathering is not fast enough to compensate for human-mediated excessive CO_2_ emissions [16]. As such, OAE has been raised as a potential mitigation tool to combat the adverse impacts of ocean acidification and climate change alike [8].

Key mineral candidates for OAE include olivine, a silicate-based mineral that occurs in magnesium-rich [forsterite (Mg_2_SiO_4_)] and iron-rich [fayalite (Fe_2_SiO_4_)] forms [17–22], and calcium-based minerals, such as slaked lime (Ca(OH)_2_), used in ocean liming strategies [12,23]. Upon dissolution, these minerals facilitate the conversion of CO_2_ into bicarbonate (HCO_3_^-^) and carbonate (CO_3_^2-^) ions, thereby increasing TA and lowering the partial pressure of CO_2_ (*p*CO_2_) in seawater [13]. This drives CO_2_ uptake from the atmosphere through air-sea gas exchange until the equilibrium is re-established.

OAE can be applied using either air-equilibrated or non-CO_2_-equilibrated approaches [24,25]. In an air-equilibrated approach, both TA and dissolved inorganic carbon (DIC) are increased simultaneously to maintain atmospheric CO_2_ equilibrium, causing minimal chemical disruption. In contrast, the non-CO_2_-equilibrated approach increases TA alone, with DIC rising gradually as atmospheric CO_2_ dissolves into the ocean and equilibrium is restored. This latter process can take weeks to months, depending on local physicochemical conditions [25–27]. While the latter approach is considered more scalable and economically feasible, it includes transient elevations in pH and declines in *p*CO_2_, which may affect biological processes [28–30]. In detail, carbonate chemistry perturbations can impact marine organisms by disrupting acid-base homeostasis [28,31] and reducing carbon availability for photosynthesis [32–37]. Moreover, primary producers could be directly impacted due to elevated ion concentrations introduced by the different alkaline substances [14,28]. For instance, iron present in olivine can support primary production as an otherwise often limiting micronutrient, similar to silicate, which is expected to benefit silicifying phytoplankton such as diatoms [28,38–41]. Such shifts in phytoplankton composition can cascade through the food webs, altering zooplankton community structure, abundance, and trophic transfer efficiency through changes in the prey availability and its overall quality.

While many studies have focused on the response of primary producers to OAE [33,34,41–46], relatively little is known about the effects of OAE on individual zooplankton species and cascading effects on plankton communities [32,47,48]. In oligotrophic and coastal systems, recent mesocosm and microcosm studies with air-equilibrated and non-CO_2_-equilibrated OAE generally report modest impacts on zooplankton standing stock and its biodiversity [47,49–51]. Laboratory and exposure studies similarly indicate that many copepod species tolerate moderate, short-term alkalinity or DIC increases, with adverse effects typically occurring only at extremely high pH levels (≥ 9–10) or strongly elevated bicarbonate conditions [37,48,52]. Together, these studies suggest that mesozooplankton can withstand a wide range of OAE perturbations, but they also highlight the lack of consideration of gelatinous zooplankton species, especially larvaceans.

Larvaceans often represent the second most abundant zooplankton group after copepods [53] and are often present in coastal ecosystems. They play a critical role in pelagic food webs, bypassing the traditional grazing pathways and directly transferring energy from picoplankton to higher trophic levels, such as fish [53]. This is achieved through their external filter structure, or ‘house’, which efficiently concentrates particles down to 0.2 µm [54]. This allows larvaceans to exploit a broad prey spectrum, including picoplankton, ciliates, bacteria, and large viruses, with a predator-to-prey size ratio of over 10,000:1 [53,55]. Once clogged, larvacean houses are discarded and can sink rapidly, scavenging surrounding particles and contributing significantly to the vertical carbon flux and biological carbon sequestration [56,57]. Additionally, larvaceans are characterized by their extraordinarily high growth rates, which are on the same order of magnitude as their microbial prey, which allows them to quickly build up biomass under favourable conditions [53]. Hence, larvaceans are key facilitators of carbon cycling and trophic transfer, particularly in systems dominated by small-sized primary producers.

Given their ecological significance, understanding the responses of larvaceans to OAE is critical. So far, experiments have investigated the resilience of larvaceans to ocean acidification [58–61]. These studies have documented that larvaceans are favoured under low pH conditions. However, their response to increased pH levels remains unknown [58–61]. To address this knowledge gap, we aimed to test how non-CO_2_-equilibrated OAE impacts (i) population dynamics of the larvacean species *Oikopleura dioica*, and (ii) the physiological performance, assessed by measuring house production and clearance rates across a ΔTA gradient of 0–600 µmol kg^-1^ using calcium‐ and silicate‐based minerals, respectively. We further assessed indirect pathways by relating larvacean densities to prey (picoplankton, chlorophyll a) and predator (>500 µm zooplankton, e.g., chaetognaths, calanoid copepods) dynamics.

We hypothesized that *O. dioica* tolerates the direct physiological stress associated with OAE-induced pH elevation, consistent with earlier reports showing no adverse effects of elevated *p*CO_2_ on its abundance or fitness [58–61]. However, such apparent tolerance often reflects indirect stimulation through enhanced food availability under acidified conditions, as Winder et al. [60] demonstrated through targeted feeding assays. Accordingly, we hypothesized that OAE-induced shifts in prey composition (bottom-up effects) would play a major role in larvacean dynamics, and that changes in predation pressure (top-down effects) under different mineral applications might further modulate population responses. Specifically, we expected higher abundances when small primary producers dominated the plankton community and when fish predator densities were low.

## Materials and methods

### Experimental design and study area

A 53-day experiment was conducted in the mesotrophic environment of Raunefjord near Bergen, Norway (60°15^’^55^”^ N, 5°12^’^21^”^ E) during the post-spring-bloom period (May-July 2022). This setting was selected, as mesotrophic conditions are typical for many temperate shelf and fjord systems, which are potential targets for nearshore OAE.

A gradient-based design was used in which TA was elevated in increments of 150 µmol per kilogram of seawater, resulting in five ΔTA levels: 0, 150, 300, 450, and 600 μmol kg^-1^ (S1 Fig) in order to test four different alkalinity levels and stay well below commonly discussed safety boundaries of ΔTA < 1000 µmol kg^-1^; pH < 9 [62]. The selected range also provides adequate resolution to detect non-linear responses and exceeds natural short-term TA variability, ensuring statistical detectability of treatment effects.

To remain within the logistical limits of large volume mesocosms, one mesocosm was assigned to each ΔTA level. This choice sacrifices treatment-level replication but provides higher gradient resolution and more closely reflects the spatial heterogeneity expected in real-world OAE deployments. This design aimed to identify safe thresholds for OAE applications with minimal ecological disruption to the planktonic community [63–66]. Furthermore, this study compared the effects of two mineral-based OAE strategies, calcium- and silicate-based applications.

Ten mesocosms, each 22 m long (∼21.5 m water column plus a 0.5 m sediment trap) with a 2 m diameter (∼ 60,000 L volume), were deployed [63]. On day 0, each mesocosm was filled with fjord water, retaining the natural planktonic community but excluding organisms > 3 mm. This truncation followed KOSMOS protocols and was applied to reduce chance-driven differences among mesocosms caused by the random enclosure of rare large predators (e.g., fish, jellyfish), which can introduce strong between-mesocosm variability and obscure treatment effects [67]. Predation pressure was instead standardized by the controlled addition of 95 laboratory-reared Atlantic herring (*Clupea harengus*) larvae per mesocosm. Mixing was passive and maintained fjord-typical vertical structure (including episodic stratification), driven primarily by wave-induced surface turbulence and convective adjustment to ambient temperature changes. Light followed ambient diel conditions. Five mesocosms were assigned to calcium-based OAE (Ca-based) treatments and five to silicate-based OAE (Si-based) treatments. On day 6, TA in both treatments was increased along a 0–600 μmol kg^-1^ gradient by adding varying amounts of sodium hydroxide (NaOH) dissolved in 20 L of Milli-Q water.

In Ca-based mesocosms, calcium chloride (CaCl_2_) was co-added in proportions equal to half the TA enhancement (i.e., Δ[Ca^2+^] = ½ ΔTA; equations 1−2). In Si-based mesocosms, magnesium chloride (MgCl_2_) was added similarly (Δ[Mg^2+^] = ½ ΔTA). To ensure that ΔTA remained the sole driver and to avoid conflating carbonate chemistry with micronutrients or particle effects, sodium metasilicate (Na_2_SiO_3_) was added at 75 µmol L^-1^ to all Si-based treatments, rather than scaled with ΔTA. A Na_2_SiO_3_ gradient would (i) create a co-gradient in Si with independent ecological effects, (ii) approach metasilicate solubility and promote colloid formation at higher doses, and (iii) increase the risk of CaCO_3_ precipitation (equation 3−6). Because each mole of Na_2_SiO_3_ contributes two equivalents of TA, the Si‐control mesocosm received 150 µmol kg^-1^ HCl to counterbalance the added TA [41,68]. All reagents were pre-dissolved and added using a vertically hauled, specially designed, and commonly used mesocosm-mixing and manipulation device [67]. First post-OAE manipulation samples were collected during the regular sampling on the following day. We acknowledge that potential slightly higher carbonate chemistry perturbations within the 1^st^ 24-hour period upon alkalinity enhancement, than those reported here.


$${\text{CaCl}}_{\text{2}}+\text{2NaOH}\to \text{Ca}(\text{OH}{)}_{\text{2}}+\text{2NaCl}$$ (1)

$$\text{Ca}(\text{OH}{)}_{\text{2}}+\text{2C}{\text{O}}_{\text{2}}\to \text{C}a^{\text{2}+}+\text{2H}{{CO}_{\text{3}}}^{-}$$ (2)

$$\text{2MgC}{\text{l}}_{\text{2}}+\text{2NaOH}\to \text{Mg}(\text{OH}{)}_{\text{2}}+\text{2NaCl}$$ (3)

$$\text{Mg}(\text{OH}{)}_{\text{2}}+\text{2C}O_{\text{2}}+\text{4}H_{\text{2}}\text{O}\to \text{MgC}O_{\text{3}}{\text{\textbackslash{}rm O}}_{\text{3}}+\text{Si}(\text{OH}{)}_{\text{4}}+\text{2H}{{CO}_{\text{3}}}^{-}$$ (4)

$$Na_{\text{2}}SiO_{\text{3}}\to \text{2}Na^{+}+Si{O_{\text{3}}}^{\text{2}-}$$ (5)

$$Ca^{\text{2}+}+Si{O_{\text{3}}}^{\text{2}-}\to CaSiO_{\text{3}}$$ (6)

The phytoplankton community was in a post-bloom stage and nutrient-limited from the start of the 53-day experiment. To alleviate the progressive nutrient limitation, establish a clear contrast between an initial nutrient-depleted phase and a subsequent nutrient-repleted phase, macronutrients were added to all mesocosms on days 26 and 28. The resulting dissolved nutrient concentrations are shown in S2 Fig.

Additional details on the study area, mesocosm infrastructure, carbonate chemistry, and the controlled addition of herring larvae are provided by Goldenberg et al. [68], Ferderer et al. [41], and Marín-Samper et al. [69].

All permits, including site access permits, were obtained in accordance with national legislation; no additional permits were required for this study.

### Sampling procedure, carbonate chemistry, nutrients, and chlorophyll a measurements

Sampling was carried out every second day throughout the experiment, followed by a consistent sequence of CTD profiling, water collection, and plankton net tows. Vertical profiles of temperatures, salinity, pH, dissolved oxygen, chlorophyll *a,* and photosynthetically active radiation (PAR) were obtained with a hand-held self-logging CTD probe (CTD60M, Sea and Sun Technologies, Trappenkamp, Germany). Immediately afterwards, depth-integrated water samples were collected with an integrating water sampler (IWS, HYDRO-BIOS, Kiel, Germany), providing a column-averaged sample from each mesocosm. Subsamples were directly taken from the IWS for parameters susceptible to gas exchange or contamination, namely dissolved inorganic nutrients (NO_3_^−^, NO_2_^−^, NH_4_, Si(OH)_4_, PO_4_^3−^), dissolved inorganic carbon (DIC), and total alkalinity (TA). For nutrient analysis, triplicate subsamples were taken to account for technical variability. These were passed through 33 mm, 0.45 µm PES membrane syringe filters (Filtropur S. SARSTEDT, Nümbrecht, Germany) within 1–2 h of collection, stored in the dark at ambient temperature, and analysed within 6 h. Concentrations of nitrate, nitrite, phosphate, and silicate were determined spectrophotometrically following Hansen and Koroleff [70], whereas ammonium was measured fluorometrically with a 10-AU fluorometer (Turner Designs, San Jose, CA, USA) according to Holmes et al. [71].

The remaining IWS water was transferred into 10 L plastic canisters, transported to the laboratory, and stored at *in situ* water temperature in a temperature-controlled room in the dark until processing later the same day. From these canisters, subsamples were taken for total particulate carbon (TPC), particulate organic carbon (POC), nitrogen (PON), total particulate phosphorus (TPP), biogenic silica (BSi), chlorophyll *a*, phytoplankton pigments, and microscopic counts of phytoplankton and microzooplankton.

For carbonate system characterization, TA, DIC, and total-scale seawater pH were measured, and the remaining carbonate parameters were calculated with CO2SYS using *in situ* temperature and salinity obtained from CTD with constants from Luecker et al. [72]. TA and pH were sampled every second day. TA was determined by potentiometric two-step titration (Metrohm 862 Compact Titrosampler, 0.05 M HCl, Aquatrode Plus Pt1000, 907 Titrando) following Chen et al. [73]. Seawater pH samples were equilibrated to 25 °C in a thermostatic bath and measured spectrophotometrically at 25 °C with a VARIAN Cary 100 and a 10 cm cuvette [74]. DIC was measured on day 9 with an AIRICA system (MARIANDA, Kiel, Germany) coupled to a LI-7000 differential gas analyser (LI-COR Biosciences GmbH, Bad Homburg, Germany) at room temperature within 12 h of sampling [75,76].

For chlorophyll *a*, 500−1000 ml subsamples were filtered onto GF/F glass fibre filters (Whatman Merck, Darmstadt, Germany), under low-light conditions. Filters were placed in plastic vials, frozen at −80°C, and analysed fluorometrically on the following day according to Welschmeyer [77].

### Larvacean abundance

Larvacean abundances were estimated along with the entire zooplankton community from samples, which were collected every four days using an Apstein net (mesh size: 55 µm, diameter: 17 cm). The net was vertically hauled to a fixed depth of 21.5 m, filtering a total volume of ~469 L per tow. The collected samples were transferred to 500 mL jars using filtered seawater and stored in a cooler during transport to the laboratory. In the laboratory, samples were size fractionated into three categories using mesh filters: small (55–200 µm), intermediate (200–500 µm), and large (>500 µm) zooplankton. Samples were preserved in 70% ethanol. Taxonomic identification and enumeration were carried out on-site using a Leica S9i stereomicroscope.

### House production and prey clearance rates estimation

On day 36, *O. dioica* specimens were collected from each mesocosm for laboratory incubation experiments to estimate house production and prey clearance rates. Surface water from each mesocosm was transferred into 5 L transparent PP beakers to individually pick *O. dioica* within their mucus houses using a wide-mouthed glass pipette. Larvaceans that showed visible gonads were excluded to avoid reproduction during incubations.

For house production rate measurements, individual larvaceans were transferred into 100 mL glass bottles pre-filled with 55 µm filtered seawater from the corresponding mesocosm. For prey clearance measurement, two 250 ml incubation bottles were prepared per mesocosm: one containing only 55 µm filtered seawater served as a control for phytoplankton growth in the absence of grazers, and the other containing three *O. dioica* individuals of similar size. All glass bottles were closed without trapping air bubbles, time was noted as the incubation start time-point, and the bottles were immersed in *in situ* seawater in a cooling container and transported to the laboratory. In the laboratory, bottles were mounted on a plankton wheel and incubated for ~24 hours in a temperature-controlled room set at 11°C, mimicking *in situ* conditions. At the end of the incubation, larvaceans were gently removed.

For the house production estimates, the removal of the animal within its house represented the termination time-point and was used to calculate house production rates throughout the experiment, expressed per hour. All *O. dioica* were individually retrieved and examined under a stereomicroscope (ZEISS Stemi 508) to measure their trunk lengths. Afterward, the incubation bottle content (100 mL) was inspected under a microscope to count all discarded houses.

For clearance rate assays, paired control and grazed bottles were run per mesocosm; both bottles were filled simultaneously from the same mesocosm water (collected using an integrated water sampler), ensuring identical initial prey concentrations. Rather than measuring initial concentrations from each bottle, we used concurrent mesocosm flow‐cytometry data, an acceptable proxy given the shared source water, to establish starting prey levels. At the end of each ~24 h incubation (11°C), phytoplankton counts were determined on a flow cytometer (BD Accuri C6). At 11°C, net phytoplankton growth typically does not exceed 0.1 d^-1^, which over a 24 h period corresponds to a < 10% change in control counts [78], so any growth‐related change in the control is small relative to grazing losses. Accordingly, we estimated apparent clearance rates using Frost’s [79] equation without a growth-correction [80], attributing the difference in final prey concentration between control and grazing bottles, assuming that any deviation from the control reflects net grazing activity.

### Data analysis

We delineated the study period into three phases based on the timing of OAE application and nutrient additions. *Phase 0* (Days 1–5) represented the pre-OAE application period. *Phase I* (Days 7–25) captured the initial responses under nutrient limitation following the OAE application. *Phase II* (Days 27–53) encompassed the post-nutrient addition period, including the subsequent repletion and eventual return to depletion. To assess top-down controls on *O. dioica*, we quantified the abundances of large zooplankton (>500 µm, e.g., copepods, chaetognaths, and hydromedusae) that are known predators of *O. dioica,* its eggs, and discarded houses [81–84].

To ensure statistical independence, daily observations were averaged within each phase, yielding one mean value per response variable per mesocosm per phase. Thus, the mesocosm was the experimental unit for all analyses. This gradient design improves our ability to detect thresholds in ΔTA responses, while maintaining ecological realism in large volume mesocosm systems [63–65].

The phase-averaged responses were analysed with Type III analysis of covariance (ANCOVA) with ΔTA as a continuous predictor (from 0 to 600 μmol kg^-1^), mineral type as a categorical predictor (silicate vs calcium), and their interaction (ΔTA × mineral). To explores potential indirect effects through trophic interactions, we computed Pearson´s correlations between *O*. *diocia* metrics and their prey and predator groups.

Variables exhibiting right skew were log_10_(x + 1) transformed before analysis; normality and homoscedasticity were confirmed by residuals vs. fitted and Q‐Q plots. All statistical tests used α = 0.05, and all analyses were performed in R (v4.4.0, R Core Team 2023).

## Results

We assessed how non-CO_2_-equilibrated OAE influences the natural plankton community, focusing on the key gelatinous zooplankton species *Oikopleura dioica* across five ΔTA treatments ranging from 0 to 600 µmol kg^-1^, using two different mineral types. *O. dioica* was the dominant gelatinous zooplankton species and the second most abundant zooplankton taxon observed throughout the experiment. No other larvaceans (e.g., *Fritillaria borealis*), which are typically present in the study area, were detected. Over the 53-day experiment, seawater pH ranged from 8.1 to 8.7 (in direct correspondence with the ΔTA treatments), and temperature increased steadily over time from 8.5 to 15.5°C in all treatments (S2 Fig).

**Fig 1:**
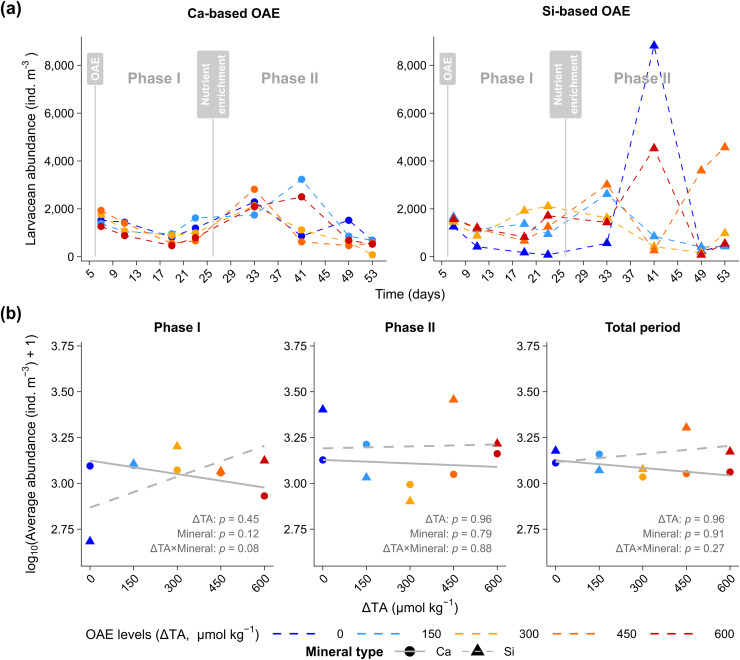
Response of larvacean (*Oikopleura dioica*) abundance to calcium (Ca) and silicate (Si) based ocean alkalinity enhancement (OAE). **(a)** Time series of *O. dioica* abundance (ind. m^-3^) in mesocosms receiving Ca-based (●) or Si-based (▲) alkalinity enhancements at different target increases in total alkalinity (ΔTA). Vertical grey lines indicate the OAE application (Day 6) and nutrient addition (Day 25). **(b)** Phase-specific relationships are shown for larvacean abundance and ΔTA levels (0–600 µmol kg^-1^) for *Phase I* (Days 7–23), *Phase II* (Days 33–53), and the total experimental period; grey lines depict linear regressions separated by mineral type into Ca-based (solid ―) and Si-based (dashed – –) treatments; symbols: Ca-based (●), Si-based (▲).

Larvacean densities varied over time without a consistent trend relative to ΔTA or mineral source (Fig 1). In *Phase I*, densities ranged from 78 to 2,106 ind. m^-3^, while in *Phase II*, abundances increased notably, particularly in the Si-based treatment. On day 41, densities ranged from 8,818 ind. m^-3^ at ΔTA 0–261 ind. m^-3^ at ΔTA 450 (Fig 1a). Despite these variations, statistical analyses detected no significant main or interactive effects of ΔTA or mineral type on larvacean abundance (*p* > 0.05; Fig 1 b).

**Fig 2:**
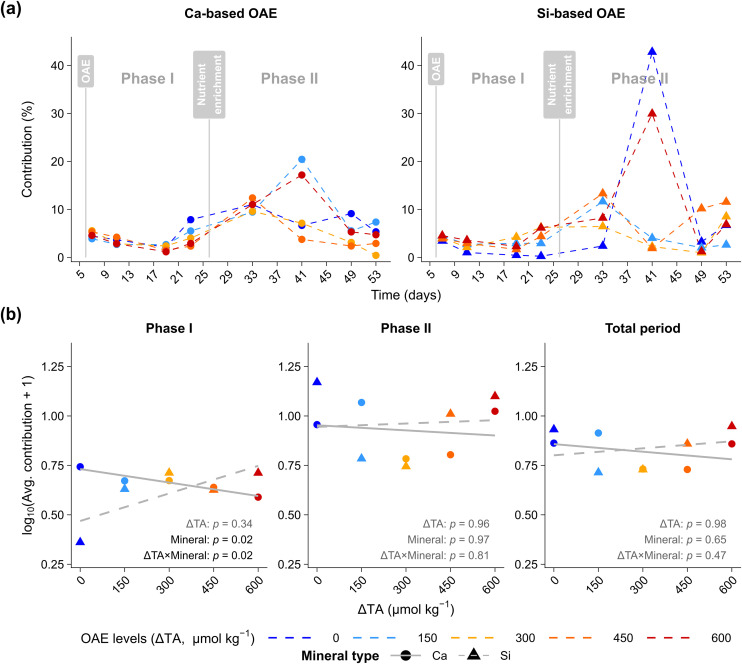
Contribution (%) of larvaceans (*Oikopleura dioica*) to the total zooplankton abundance under calcium (Ca) and silicate (Si) based ocean alkalinity enhancement (OAE). **(a)** Time series of the percentage contribution of *O. dioica* to total zooplankton abundance in mesocosms receiving Ca-based (●) or Si-based (▲) alkalinity enhancements at different target increases in total alkalinity (ΔTA). Vertical grey lines indicate the OAE application (Day 6) and nutrient addition (Day 25). **(b)** Phase-specific relationships between contribution (%) and ΔTA levels (0–600 µmol kg^-1^) for *Phase I* (Days 7–23), *Phase II* (Days 33–53), and the total period, with grey lines depict linear regressions separated by mineral type into Ca-based (solid —) and Si-based (dashed – –) treatments; symbols: Ca-based (●), Si-based (▲).

Similarly, the relative contribution of larvaceans to the total zooplankton community abundance varied across phases but showed no clear response to ΔTA or mineral type (Fig 2). Contributions in Si-based treatments ranged from 0.5% to 5.6% in *Phase I*. *Phase II* exhibited greater variability, with a maximum of 42.8% in the control (ΔTA 0) and 30.0% at the highest alkalinity treatment with ΔTA 600. In contrast, Ca-based treatments showed an overall lower contribution of larvaceans to the mesozooplankton community, ranging from 0.43% (ΔTA 300) to 20.44% (ΔTA 150; Fig 2a). A significant ΔTA × mineral interaction was observed in *Phase I* (*p* = 0.02), probably driven by the extremely low contribution of larvaceans to the mesozooplankton community in the silicate control treatment throughout *phase I* (Fig 2b). However, no persistent effects were detected in *Phase II* or over the entire investigation period (Fig 2b).

**Fig 3:**
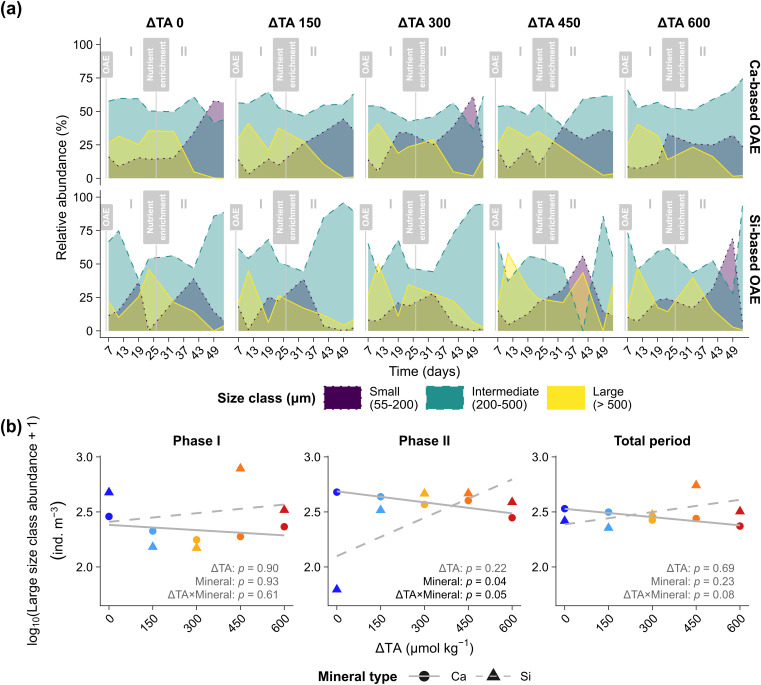
Size‐distribution responses of larvacean (*Oikopleura dioica)* under calcium (Ca) and silicate (Si) based ocean alkalinity enhancement (OAE). **(a)** Temporal dynamics of the relative abundances (%) of three larvacean size classes: small (55–200 µm), intermediate (200–500 µm), and large (>500 µm) across ΔTA treatments (0–600 µmol kg^-1^). Vertical grey lines indicate the OAE application (Day 6) and nutrient addition (Day 25). **(b)** Phase specific relationship between the abundance (ind. m^-3^) of the large size class and ΔTA levels for *Phase I* (Days 7–23), *Phase II* (Days 33–53), and the total period; with grey lines depict linear regressions separated by mineral type into Ca-based (solid —) and Si-based (dashed – –) treatments; symbols: Ca-based (●), Si-based (▲).

We then examined changes in the size distribution of the larvacean community by categorizing individuals as small (55–200 µm), intermediate (200–500 µm), and large (> 500 µm). In Ca-based treatments, intermediate size classes dominated *Phase I* (>50%), large individuals contributed 34.1% (Day 19; ΔTA 300), while small individuals were rare. In *Phase II*, small individuals contributed 58.2% (Day 49; ΔTA 0), while large size classes declined (<3%) and ultimately disappeared on Day 53. Si-based treatments showed parallel trends, with intermediate sizes dominating and rising from 74.8% (Day 11; ΔTA 0) to 95.8% (Day 53; ΔTA 600). In contrast, large individuals declined, and small-sized larvaceans appeared only briefly and peaked at ΔTA 600 (69.4%) on Day 49 (Fig 3a). Despite these variations, statistical analyses did not detect an effect of ΔTA or mineral type on small or intermediate classes. However, a significant effect was detected for ΔTA × mineral interaction (*p* = 0.05; Fig 3b) for large larvaceans in *phase* *II*. These patterns likely reflect timing and population turnover, rather than a direct physiological impact due to alkalinity changes.

**Fig 4:**
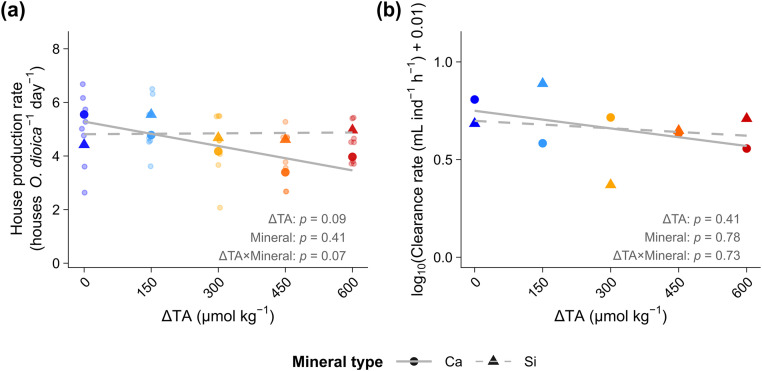
Larvacean (*Oikopleura dioica*) house production and prey-clearance rates in response to calcium (Ca) and silicate (Si) based ocean alkalinity enhancement (OAE). **(a)** House production rate (houses ind.^-1^ d^-1^) and **(b)** prey-clearance rates (mL ind.^-1^ h^-1^) of *O*. *dioica* measured on Day 36 under Ca-based (●) and Si-based (▲) OAE across ΔTA treatments (0–600 µmol kg^-1^). Grey lines depict linear regressions separated by mineral type into Ca-based (solid —) and Si-based (dashed – –) treatments.

To assess direct effects on larvacean physiology, we measured house production and prey-clearance rates in incubation assays on Day 36 (*Phase II*; Fig 4). No significant effects were observed on either house production or feeding rates (clearance rates) across the ΔTA gradient and mineral types. While mean rates appeared slightly lower in Ca-based treatments, the differences were not statistically significant due to high variability (*p* > 0.05; Fig 4a-b).

**Fig 5:**
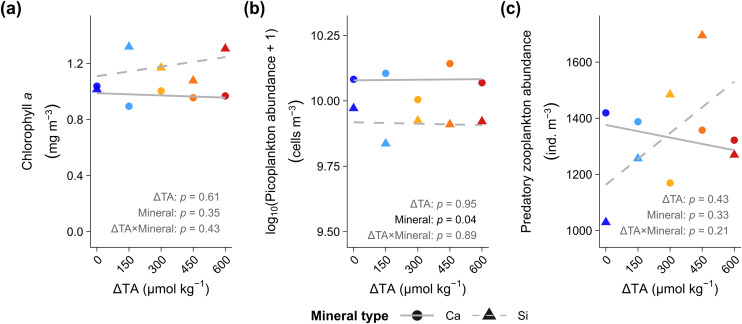
Influence of ocean alkalinity enhancement (OAE) on potential indirect (trophic) pathways that could influence larvacean (*Oikopleura dioica).* Relationship between average (**a)** total primary producers (chlorophyll *a*, mg m^-3^), **(b)** primary food source (picoplankton, cells m^-3^), and **(c)** predators (zooplankton >500 μm; ind. m^-3^) over Days 7−53 and ΔTA (0–600 µmol kg^-1^) across Ca-based (●) and Si-based (▲) treatments. Grey lines depict linear regressions separated by mineral type into Ca-based (solid —) and Si-based (dashed – –) treatments.

To test indirect pathways, we examined changes in both prey (chlorophyll *a*; picoplankton) and predator (>500 μm zooplankton, including calanoid copepods, chaetognaths, and hydromedusa) over time (S5 Fig) and, whether these have been directly impacted by OAE (Fig 5). Chlorophyll *a* remained unchanged across ΔTA treatments, indicating no direct impact on overall primary production (Fig 5a). In contrast, picoplankton exhibited mineral-driven differences, with Ca-based treatments exhibiting slightly but consistently higher abundances (*p* = 0.05; Fig 5b). Additionally, temporal peaks in picoplankton often preceded larvacean blooms, suggesting bottom-up control (S5a Fig). For instance, in *Phase II*, the peak larvacean abundance in control Si-based treatment coincided with a prior increase in picoplankton (S5b Fig). Similarly, delayed chlorophyll *a* maximum in specific treatments (ΔTA 0 and 600 in Si-based, ΔTA 150 and 600 in Ca-based) were followed by elevated larvacean densities (S5a Fig), consistent with the rapid larvacean responses to increased food availability in primary and microbial food resources following nutrient input. Predator abundances showed no systematic response to OAE (Fig 5c).

**Fig 6:**
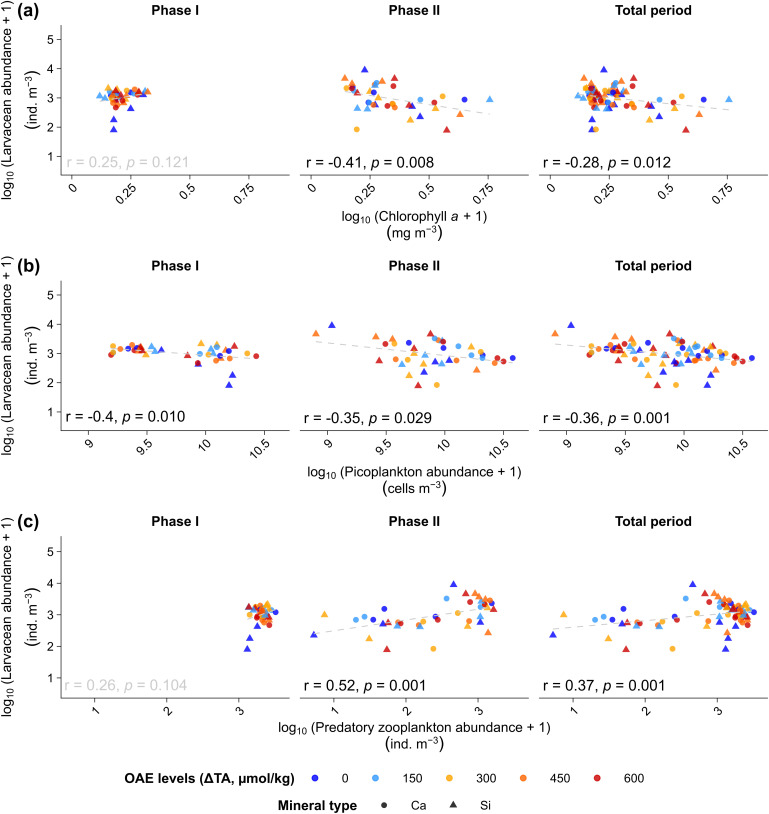
Indirect (trophic) influences of ocean alkalinity enhancement (OAE) on larvacean (*Oikopleura dioica).* Pearson correlations between O. *dioica* abundance (ind. m^-3^) and **(a)** chlorophyll *a* concentration (mg m^-3^), **(b)** picoplankton abundance (cells m^-3^), and **(c)** predatory zooplankton abundance (>500 μm; ind. m^-3^) for *Phase I* (Days 7–23), *Phase II* (Days 33–53), and the total experimental period across Ca-based (●) and Si-based (▲) treatments. Dashed regression lines show fitted linear relationships.

To determine whether OAE altered larvacean grazing pressure on primary producers and picoplankton, we conducted Pearson correlation analyses between *O. dioica* abundance and key prey metrics. During *Phase I*, there was a weak positive correlation with chlorophyll *a (r = 0.25, p = 0.12),* suggesting limited grazing impact or prey control at early stages. In *Phase II*, however, a significant negative correlation emerged (*r* = −0.41, *p* = 0.008), consistent with intensified grazing following nutrient enrichment. This negative association persisted over the total experimental period (*r* = −0.28, *p* = 0.01; Fig 6a). Likewise, in individual phases and over the total period (*r* = −0.36, *p* = 0.001; Fig 6b), significant negative correlations between larvacean and picoplankton were observed, suggesting sustained top-down control on picoplankton by larvacean.

Finally, to test for predator-mediated effects on larvaceans, we examined correlations between larvacean and predator abundances. Contrary to expectations of inverse relationships, we observed significant positive correlations in *Phase II* (*r* = 0.52, *p* = 0.001) and across the full study period (*r* = 0.37, *p* = 0.001; Fig 6c). These concurrent increases likely reflect shared responses to increased resource availability following nutrient enrichment, supporting elevated abundances across multiple zooplankton groups during this phase.

## Discussion

### Resilience of *Oikopleura dioica* to OAE

Our 53‐day mesocosm study demonstrated that the larvacean *O. dioica* sustained stable population densities and key physiological functions (house production, prey clearance) under non-CO_2_-equilibrated OAE conditions across a ΔTA gradient up to 600 µmol kg^-1^ (ΔpH 0.7). Despite notable temporal variability, no consistent effect of alkalinity enhancement was detected. Although overall responses were similar across Ca- and Si-based treatments, we observed a transient effect of ΔTA-mineral interactions on the larger larvacean size fraction. Marín‐Samper et al. [69] reported delayed phytoplankton bloom onset and reduced community production in Ca‐enhanced mesocosms. Ferderer et al. [41] found enhanced diatom silicification under Si‐based OAE. Collectively, these studies highlight that alkalinity source, beyond its direct contribution to TA, can modulate ecological outcomes by modulating the phytoplankton community. In addition, we observed correlations between larvacean abundance and both picoplankton and predator groups; these must be viewed in light of the mesocosm’s overlapping biotic and abiotic processes. Our study was not designed to disentangle food web interactions from direct chemical effects. Nevertheless, collectively, these results underscore the resilience of *O. dioica* to the tested OAE scenarios, contributing to our understanding of zooplankton responses to this mCDR approach. Due to our mesocosm set-up, we cannot differentiate direct pH/TA effects (e.g., acid-base disruption) from indirect, trophic-mediated responses (e.g., shifts in prey abundance or quality, predator composition). Quantifying each pathway independently was therefore beyond this study’s scope, but this design reflects real-world OAE deployments, where chemical perturbations and ecological interactions co-occur. To delineate our interpretation clearly, we have organized the discussion into two complementary sections: first, evaluating the plausibility of direct physiological impacts of elevated pH on *O. dioica*, and second, examining how treatment-driven changes in prey and predator dynamics could have indirectly shaped larvacean population patterns.

### Potential direct physiological effects of elevated pH

Key functional traits, house production and prey clearance rates, remained stable across the ΔTA gradient (0–600 µmol kg^-1^) and both mineral treatments, demonstrating *O. dioica*’s capacity to maintain energetically demanding processes under elevated pH (8.1–8.7). This finding extends prior observations of larvacean resilience under acidified conditions [58–60], and now extends this tolerance to more alkaline conditions. Nonetheless, it is important to note that both house production and clearance rates are energetically demanding processes that can be sensitive to environmental stressors, including pH shifts. For example, Bouquet et al. [58] used microcosm life-cycle experiments (~5–6 days from fertilization to spawning) and showed that exposure to elevated pH (≥ 8.4) delayed reproduction by ~2–5 h and significantly reduced egg production, leading to a lower population growth rate (r_max_) in *O*. *dioica*. Similarly, pH‐driven alterations in biochemical pathways could impair mucus synthesis and structural integrity of the house, although such effects may only emerge under more extreme or prolonged exposures and require further investigation.

Although our incubation experiment revealed no significant impact, subtle or delayed effects at elevated pH remain plausible, particularly under food‐limited or in a multi‐stressor context. Notably, our mesocosm data also did not indicate adverse population-level effects. Some of the highest larvacean densities were observed at the highest ΔTA treatments, particularly in Si-based additions. Although this could suggest tolerance or even a positive association with elevated alkalinity, these patterns likely reflect ecological interactions, such as nutrient-driven prey availability, rather than direct chemical stimulation by pH.

### Indirect effects through trophic interactions

Although *O. dioica* density did not vary with ΔTA, its population dynamics appear to have been shaped by food-web-mediated interactions. Larvacean abundance closely tracked temporal changes in prey availability, picoplankton abundance, which itself differed among Ca- and Si-based treatments. For instance, the sharp increase in *O. dioica* density observed at ΔTA 0 µmol kg^-1^ in the Si-based treatment during *Phase II* coincided with a prior rise in picoplankton, suggesting a food-mediated response following nutrient enrichment, as previously observed in a mesocosm set-up [85].

We also recorded shifts in larvacean size structure; larger individuals became more prevalent due to ΔTA-mineral interaction. Such trait shifts are also likely food-mediated, as previous studies have shown that *O. dioica* matures at smaller sizes under low food conditions, with maturation sizes ranging between 600 and 1600 µm, depending on resource levels [86]. Thus, the observed differences in size distribution may reflect indirect responses to changing trophic conditions rather than direct physiological effects of OAE. Specifically, picoplankton was impacted differently due to the mineral type. Such trait-level shifts highlight the importance of considering indirect ecological pathways when assessing the impacts of OAE.

Our study period followed the spring bloom, a period characterized by low macronutrient concentrations and a shift toward smaller phytoplankton [87]. These conditions are favourable for larvaceans, which can efficiently exploit a broad prey size spectrum, including picoplankton [88], which was more prevalent during the early phase of this study. While nutrient addition in *Phase II* increased overall microbial food availability, the relative contribution of larvaceans to the total zooplankton community also increased. However, this shift likely reflects not only increased larvacean growth but also could be due to a decline in copepod abundance, which may be driven by predation from fish larvae present in the mesocosms [59]. Additionally, it has been documented that larvaceans can contribute relatively more to secondary production under eutrophic conditions [88,89], which is related to their extraordinary growth capacity, following the growth rates of their protozoan prey [53]. Hence, without heavy predation, their ability to utilize prey resources is directly translated into positive population growth rates. The observed reduction of chlorophyll *a* in treatments with high larvacean abundance further illustrates how prey exploitation by *O. dioica* can influence phytoplankton dynamics and, by extension, impact phytoplankton standing stocks. Earlier work documented strong top-down control of larvaceans. Tönnesson et al. [90] reported that in Gullmar Fjord, *O. dioica* removed ~0.06% d^-1^ of phytoplankton (0.4% d ⁻ ^1^ bacteria), while Sato et al. [91] observed that larvacean peaks coincided with reduced chlorophyll a, and *O. dioica* removed 0.05–5% d^-1^ particulate organic carbon (POC) in eutrophic Tokyo Bay waters. Mechanistically, Scheinberg et al. [92] reported that larvaceans efficiently clear pico- and nanoplankton, and López-Urrutia et al. [93] estimated that larvaceans can account for up to ~10% d^-1^ phytoplankton removal and ~40% of total mesozooplankton grazing, consistent with the chlorophyll reduction observed here under high larvacean abundance.

As efficient suspension feeders, *O. dioica* can exert substantial grazing pressure on microbial prey [88]. Our observed negative correlations between larvacean dynamics and picoplankton, particularly during *Phase II*, are in line with this mechanism. The potential for OAE to influence not only prey quantity but also prey quality warrants attention. Additionally, the temporal patterns support *O. dioica*’s known ability to rapidly respond to changes in food availability, due to its short generation times and high reproductive rates [53,58,94].

Phytoplankton responses to pH elevation remain a critical consideration for trophic interactions. The highest pH observed in our study (8.7) falls within a range where influence on phytoplankton growth has been documented. For example, Oberlander et al. [34] found that approximately half of the coastal phytoplankton taxa experienced reduced growth at pH > 8.5, while other studies reported declines at pH ≥ 9 (ΔTA ≥ 500 µmol kg^-1^) [32,33]. These results highlight taxon-specific sensitivities and raise the possibility that prey availability to *O. dioica* may have been modulated under higher OAE treatments in our study. However, the nutrient-limited nature of our experimental system likely constrained the expression of a pronounced OAE response [69]. In oligotrophic environments, nutrient availability might have exerted greater influence on prey communities than alkalinity perturbations. This emphasizes the importance of accounting for regional nutrient regimes and seasonal dynamics when assessing OAE effects. Although the nutrient additions triggered an overall phytoplankton bloom, and Marín‐Samper et al. [69] reported that higher TA can delay bloom onset, this bloom did not emerge until the experiment’s final phase. As a result, we could not detect any cascading effects of bloom timing on *O. dioica* within our study window. Moreover, elevated pH can influence prey quality. Paul et al. [95] reported a modest decline in particulate organic nitrogen and a concurrent rise in the C:N ratio of particulate matter. Such changes can reduce the nutritional quality of prey for filter feeders like *O. dioica*. Additionally, experimental evidence suggests that elevated pH reduces the digestibility of phytoplankton, with consequences for larvacean feeding efficiency and fecundity [58]. Given its reliance on microbial prey, high metabolic demands, and limited energy reserves [96–100].

Finally, we observed positive associations between larvaceans and larger zooplankton (>500 μm), such as copepods, chaetognaths, and hydromedusa. This likely reflects a shared response to nutrient enrichment but could also indicate a shared vulnerability to fish predation, rather than a direct relation to predation-prey interactions among zooplankton. While our data did not indicate strong predation-driven suppression of the larvacean community, we cannot exclude top-down effects, especially from fish predators whose assemblage was not comprehensively characterized throughout the experiment [68]. Combined laboratory and mesocosm experiments demonstrated that early-stage fish (Atlantic herring larvae) were physiologically resilient to OAE conditions, with no significant impacts on either survival or growth [68]. However, alterations in the community composition were observed, particularly under Ca-based OAE treatments. While overall fish biomass increased, these community-level shifts may have modulated predator-prey dynamics and influenced top-down control on the zooplankton population. Previous studies have shown that copepods, medusae, ctenophores, chaetognaths, and other predators can exert substantial pressure on larvacean eggs and juveniles [82,85,89,101], especially under nutrient-depleted conditions when feeding preferences shift. For instance, predation on larvaceans might intensify due to shifts in the feeding preferences of omnivore copepods as well as an expected increase in larger-sized calanoid copepods that are known for their grazing impact on larvacean communities [101]. In summary, although we did not detect consistent trophic cascades, this may be due to the limited sampling resolution and incomplete characterization of predators.

### Outlook and implications

The ecological viability of OAE depends not only on its carbon sequestration efficacy but also on its compatibility with marine food webs, biodiversity, and ecosystem functioning. Our findings indicate that *O. dioica*, due to its physiological resilience and capacity for rapid exploitation of available prey, could continue to play a stabilizing role in the zooplankton community under these tested non-CO_2_-equilibrated ΔTA conditions. However, defining operationally realistic ΔTA levels for scalable OAE remains an open question [102], because there is still a gap between small-scale field trials and high-dose experimental studies. For example, Guo et al. [49] tested low ΔTA increases (16−29 µmol kg^-1^) using NaOH, olivine, and steel slag, during shipboard incubations in the Equatorial Pacific. They reported limited phytoplankton impacts aside from subtle shifts in cyanobacteria and picoeukaryotes. In contrast, Bednaršek et al. [103] synthesized calcification responses across 84 marine species and identified biological thresholds between 50 and 500 µmol kg^-1^, where calcification rates declined by 50%. Their findings highlight that ecological responses can emerge well before extreme pH or TA levels are reached, which highlights the need for more dedicated mechanistic investigations.

Within this context, our mesocosm experiment deliberately extended ΔTA up to 600 µmol kg^-1^ to identify potential tipping points. While this magnitude likely exceeds what many operational OAE deployments would target, it falls within proposed regulatory maxima, such as the Isometric protocol’s recommendation to remain below ΔTA ~ 1000 µmol kg^-1^ and pH 9, to prevent runaway precipitation and to limit impacts on sensitive marine organisms [62]. The high ΔTA levels in our study are best interpreted as approximating conditions near the centre of an alkalinity discharge plume, where perturbations are most intense, but would be rapidly diluted at larger scales [50]. Such high-exposure tests are valuable for delineating ecological resilience margins, identifying early-warning indicators of ecosystem disruption, and constraining safe-operating spaces for real-world OAE applications.

To refine safe-operating spaces and improve ecological risk assessment for scalable OAE, future experiments should (i) test particulate OAE sources side-by-side with dissolved additions; (ii) add trace-metal characterization and controlled particle loads to isolate chemical vs particulate effects; (iii) study across seasons to capture impacts with changing nutrient supply; (iv) include higher sampling frequency to resolve short-lived predator-prey interactions; and (v) directly quantify larvacean -mediated export using sediment or gel traps and *in situ* sensors.

Ultimately, it needs to be considered that real-world OAE deployments will occur in a dynamic and multi-stressor-impacted natural environment. Hence, interactions between warming, deoxygenation, and nutrient regimes may lead to synergistic or antagonistic interactions [104]. To support the responsible scaling of OAE, future research should employ factorial, long-term experiments that integrate diverse trophic levels and examine key organismal traits beyond abundance, such as reproduction and metabolic plasticity.

## Conclusion

Our mesocosm study shows that the gelatinous zooplankton species *Oikopleura dioica* can be resilient to non-CO_2_-equilibrated ocean alkalinity enhancement using calcium (slaked lime) and silicate (olivine) based mineral types across operational ΔTA levels up to 600 µmol kg^-1^ (pH ≤ 8.7). Key physiological functions such as house production, feeding (prey clearance), and population turnover were maintained under elevated alkalinity, suggesting that larvacean-mediated particulate export and vertical carbon flux processes are unlikely to be impaired within the tested OAE scenarios and ranges. While our gradient design was not intended to disentangle indirect trophic pathways from direct pH effect, observed temporal variations in *O. dioica* population dynamics could potentially be linked to shifts in picoplankton availability. However, a higher sampling frequency is needed to detect these detailed predator-prey dynamics. Our findings underscore the importance of food-web-level assessments when evaluating OAE as a viable mCDR strategy.

## Supporting information

S1 Fig Experimental design of the mesocosm study. Schematic of the ten KOSMOS mesocosms and sampling schedule. **(a)** shows the mesocosm structure with a floating frame, mesocosm bag (water column), and sediment trap. Layout of the two mineral-based OAE scenarios. Five Ca-based (slaked lime) mesocosms and five Si-based (olivine) mesocosms were assigned to a non-CO_2_-equilibrated ΔTA gradient of 0, 150, 300, 450, and 600 µmol kg^-1^. Circles indicate Ca-based treatments and triangles Si-based treatments; marker color denotes ΔTA level. **(b)** Sampling schedule over the 53-day experiment. Blue drops indicate CTD casts and integrated water sampling, and the plankton net symbol indicates zooplankton net hauls. The timing of OAE addition (Day 6), nutrient enrichments (Days 26 and 28), and the two main phases (*Phase I* and *Phase II*) are indicated.
(PNG)

S2 Fig Temporal development of (a) temperature (°C), (b) total alkalinity (TA; µmol kg^-1^), (c) pH (total scale), and (d) pCO_2_ (µatm) during the mesocosm experiment. The vertical grey line marks the day of ocean alkalinity enhancement (OAE) application (Day 6) and subsequent nutrient enrichment (Day 25).
(TIFF)

S3 Fig Temporal variations of dissolved nutrient concentrations during the mesocosm experiment: (a) silicate (Si(OH)_4_; µmol L^-1^), (b) nitrate (NO_3_^-^; µmol L^-1^), (c) nitrite (NO_2_^-^; µmol L^-1^), and (d) phosphate (PO_4_^3-^; µmol L^-1^) in Ca- and Si-based OAE treatments across ΔTA levels. The vertical grey line marks the day of ocean alkalinity enhancement (OAE) application (Day 6) and subsequent nutrient enrichment (Day 25).
(TIFF)

S4 Fig Larvacean (*Oikopleura dioica)* abundances in small (a) and intermediate (b) size classes as a function of total alkalinity increase (ΔTA) during each experimental phase. Data are shown as averages for *Phase I* (Days 7–25), *Phase II* (Days 26–53), and the combined period.
(TIFF)

S5 Fig Temporal variation in (a) chlorophyll *a* concentration, (b) picoplankton abundance, and (c) predatory zooplankton abundance, including copepods, chaetognaths, and hydromedusae. (TIFF)

S6 Fig Relationships between larvacean abundance (m^-3^) and sediment-trap export material (dry weight, 48h^-1^) expressed as log_10_ (x + 1) for *Phase I*, *Phase II*, and the total experimental period based on Pearson´s correlation analysis. (TIFF)

S1 File **Table S1.** Type III ANCOVA results for *Oikopleura dioica* abundance (response variable: log_10_(total abundance + 1); see Fig 1b) in relation to ocean alkalinity enhancement (OAE). ΔTA (µmol kg^-1^) was treated as a continuous predictor, and mineral type (Ca-based vs. Si-based) as a categorical factor. MS, mean square; df, degrees of freedom; F, F statistic; *p*, p value. Significance threshold α = 0.05. **Table S2.** Type III ANCOVA results for the contribution of *Oikopleura dioica* to total zooplankton abundance (%) under OAE (see Fig 2b). ΔTA was included as a continuous covariate and Mineral as a fixed factor. MS, mean square; df, degrees of freedom; F, F statistic; *p*, p value. Significance threshold α = 0.05. **Table S3.** Type III ANCOVA examining the effects of total alkalinity increase (ΔTA, continuous) and alkalinity source mineral (categorical) on three larvacean size classes (small, intermediate, large). Results correspond to Fig 3b and Supplementary Figs S1a-b. MS, mean square; df, degrees of freedom; F, F statistic; *p*, p value. Significance threshold α = 0.05. **Table S4.** Type III ANCOVA examining the effects of total alkalinity increase (ΔTA, continuous) and alkalinity source mineral (categorical) on the house production and clearance rates of *Oikopleura dioica* under OAE. Results correspond to Figs 4a-b. MS, mean square; df, degrees of freedom; F, F statistic; *p*, p value. Significance threshold α = 0.05. **Table S5.** Type III ANCOVA examining the effects of total alkalinity increase (ΔTA, continuous) and alkalinity source mineral (categorical) on prey availability and predatory zooplankton abundance of larvacean under OAE. Results correspond to Figs 5a-c. MS, mean square; df, degrees of freedom; F, F statistic; *p*, p value. Significance threshold α = 0.05.
(DOCX)
