# Correction: Resilience of the gelatinous zooplankton species *Oikopleura dioica* to ocean alkalinity enhancement

**DOI:** 10.1371/journal.pone.0351317

**Published:** 2026-06-08

**Authors:** The PLOS One Staff

There are errors in the author affiliations. The correct affiliations are as follows:

Amrita Bhaumik^1,2^, Nicolás Sánchez^3^, Silvan Urs Goldenberg^3^, Synne Spjelkavik^4^, María Couret^5^, Ulf Riebesell^3^, Maarten Boersma^1,6,7^, Cornelia Jaspers^4^

**1** Alfred-Wegener-Institut Helmholtz-Zentrum für Polar- und Meeresforschung, Biologische Anstalt Helgoland, Helgoland, Germany, **2** Department of Coastal Systems, Royal Netherlands Institute for Sea Research (NIOZ), Den Burg, the Netherlands, **3** GEOMAR Helmholtz Centre for Ocean Research Kiel, Kiel, Germany, **4** Centre for Gelatinous Plankton Ecology and Evolution, DTU Aqua – Technical University of Denmark, Lyngby, Denmark, **5** Instituto de Oceanografíay Cambio Global (IOCAG), Universidad de Las Palmas de Gran Canaria, Unidad Asociada, Universidad de Las Palmas de Gran Canaria-Consejo Superior de Investigaciones Científicas (ULPGC-CSIC), Telde, Spain, **6** Fachbereich 2 Biologie/Chemie, Universität Bremen, Bremen, Germany, **7** Alfred-Wegener-Institut Helmholtz-Zentrum für Polar- und Meeresforschung, Wadden Sea Station, Sylt, Germany.

The following information is missing from the Funding statement: The authors acknowledge support by the Open Access publication fund of Alfred-Wegener-Institut Helmholtz-Zentrum für Polar- und Meeresforschung.

The publisher apologizes for the errors.
