# Supplementary figures and images for: Resilience of the gelatinous zooplankton species Oikopleura dioica to ocean alkalinity enhancement
Source: PLoS One. 2026 Mar 30;21(3):e0344503. doi: 10.1371/journal.pone.0344503 (PMC13035162; doi:10.1371/journal.pone.0344503)

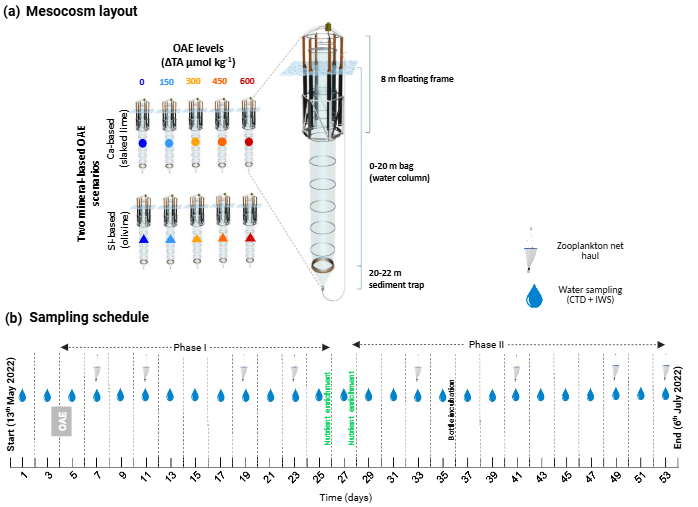

Supplement: S1 Fig — Schematic of the ten KOSMOS mesocosms and sampling schedule. (a) shows the mesocosm structure with a floating frame, mesocosm bag (water column), and sediment trap. Layout of the two mineral-based OAE scenarios. Five Ca-based (slaked lime) mesocosms and five Si-based (olivine) mesocosms were assigned to a non-CO2-equilibrated ΔTA gradient of 0, 150, 300, 450, and 600 µmol kg-1. Circles indicate Ca-based treatments and triangles Si-based treatments; marker color denotes ΔTA level. (b) Sampling schedule over the 53-day experiment. Blue drops indicate CTD casts and integrated water sampling, and the plankton net symbol indicates zooplankton net hauls. The timing of OAE addition (Day 6), nutrient enrichments (Days 26 and 28), and the two main phases (Phase I and Phase II) are indicated. (PNG) [file pone.0344503.s001.png]

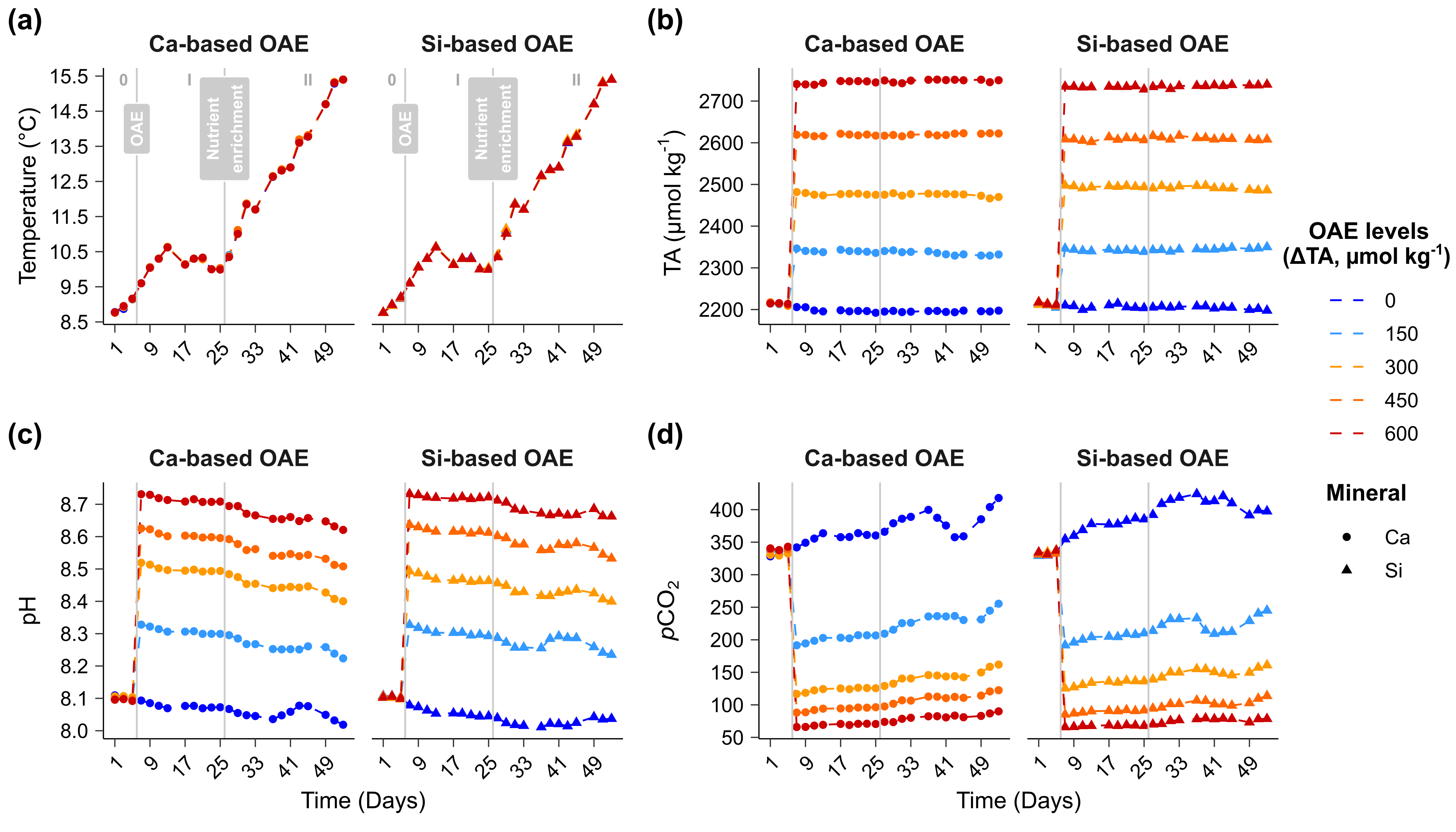

Supplement: S2 Fig — The vertical grey line marks the day of ocean alkalinity enhancement (OAE) application (Day 6) and subsequent nutrient enrichment (Day 25). (TIFF) [file pone.0344503.s002.tiff]

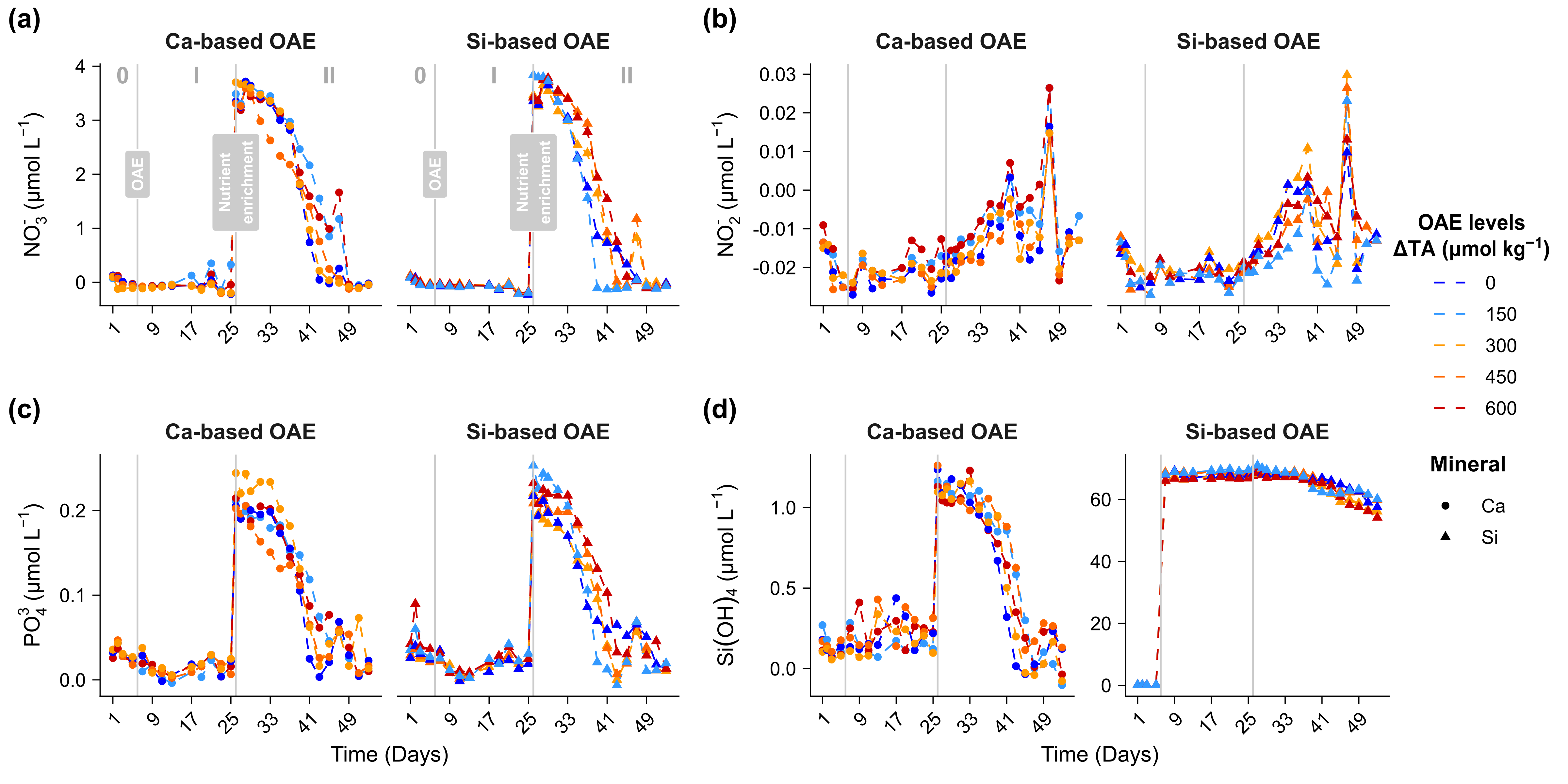

Supplement: S3 Fig — The vertical grey line marks the day of ocean alkalinity enhancement (OAE) application (Day 6) and subsequent nutrient enrichment (Day 25). (TIFF) [file pone.0344503.s003.tiff]

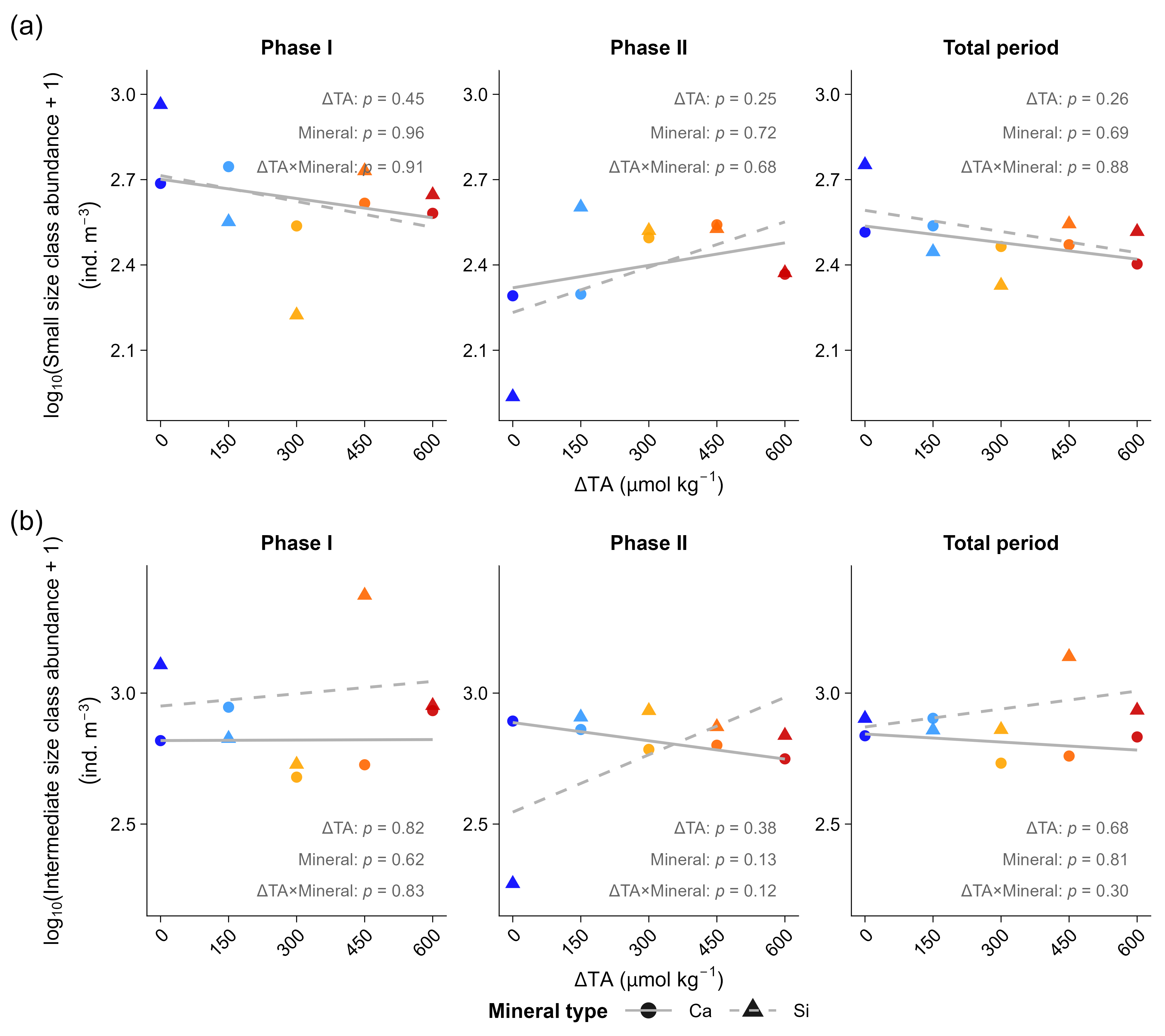

Supplement: S4 Fig — Data are shown as averages for Phase I (Days 7–25), Phase II (Days 26–53), and the combined period. (TIFF) [file pone.0344503.s004.tiff]

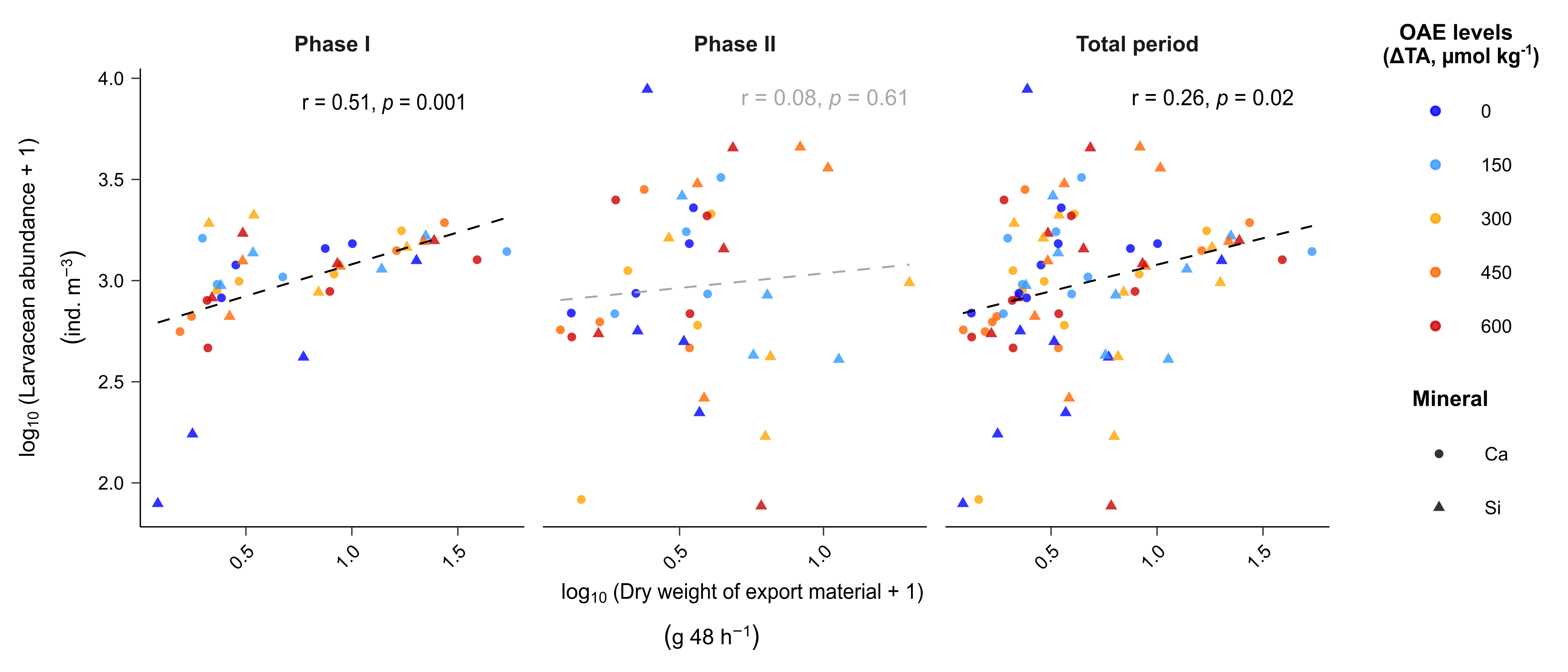

Supplement: S6 Fig — (TIFF) [file pone.0344503.s006.tiff]
