# Supplementary material for: Resilience of the gelatinous zooplankton species Oikopleura dioica to ocean alkalinity enhancement
Source: PLoS One. 2026 Mar 30;21(3):e0344503. doi: 10.1371/journal.pone.0344503 (PMC13035162; doi:10.1371/journal.pone.0344503)
Supplement: S1 File — Table S1. Type III ANCOVA results for Oikopleura dioica abundance (response variable: log10(total abundance + 1); see Fig 1b) in relation to ocean alkalinity enhancement (OAE). ΔTA (µmol kg-1) was treated as a continuous predictor, and mineral type (Ca-based vs. Si-based) as a categorical factor. MS, mean square; df, degrees of freedom; F, F statistic; p, p value. Significance threshold α = 0.05. Table S2. Type III ANCOVA results for the contribution of Oikopleura dioica to total zooplankton abundance (%) under OAE (see Fig 2b). ΔTA was included as a continuous covariate and Mineral as a fixed factor. MS, mean square; df, degrees of freedom; F, F statistic; p, p value. Significance threshold α = 0.05. Table S3. Type III ANCOVA examining the effects of total alkalinity increase (ΔTA, continuous) and alkalinity source mineral (categorical) on three larvacean size classes (small, intermediate, large). Results correspond to Fig 3b and Supplementary Figs S1a-b. MS, mean square; df, degrees of freedom; F, F statistic; p, p value. Significance threshold α = 0.05. Table S4. Type III ANCOVA examining the effects of total alkalinity increase (ΔTA, continuous) and alkalinity source mineral (categorical) on the house production and clearance rates of Oikopleura dioica under OAE. Results correspond to Figs 4a-b. MS, mean square; df, degrees of freedom; F, F statistic; p, p value. Significance threshold α = 0.05. Table S5. Type III ANCOVA examining the effects of total alkalinity increase (ΔTA, continuous) and alkalinity source mineral (categorical) on prey availability and predatory zooplankton abundance of larvacean under OAE. Results correspond to Figs 5a-c. MS, mean square; df, degrees of freedom; F, F statistic; p, p value. Significance threshold α = 0.05. (DOCX) [file pone.0344503.s007.docx]

**Supplementary material**

**Resilience of the gelatinous zooplankton species *Oikopleura diocia* to ocean alkalinity enhancement**

Bhaumik et al.

Correspondence to: Amrita Bhaumik ([amrita.bhaumik@nioz.nl](mailto:amrita.bhaumik@nioz.nl)) and Cornelia Jaspers ([coja@aqua.dtu.dk](mailto:coja@aqua.dtu.dk))

**Fig S1.** Experimental design of the mesocosm study. Schematic of the ten KOSMOS mesocosms and sampling schedule. **(a)** shows the mesocosm structure with a floating frame, mesocosm bag (water column), and sediment trap. Layout of the two mineral-based OAE scenarios. Five Ca-based (slaked lime) mesocosms and five Si-based (olivine) mesocosms were assigned to a non-CO_2_-equilibrated ΔTA gradient of 0, 150, 300, 450, and 600 µmol kg^-1^. Circles indicate Ca-based treatments and triangles Si-based treatments; marker color denotes ΔTA level. **(b)** Sampling schedule over the 53-day experiment. Blue drops indicate CTD casts and integrated water sampling, and the plankton net symbol indicates zooplankton net hauls. The timing of OAE addition (Day 6), nutrient enrichments (Days 26 and 28), and the two main phases (Phase I and Phase II) are indicated.

**Fig S2.** Temporal development of **(a)** temperature (°C), **(b)** total alkalinity (TA; µmol kg^-1^), **(c)** pH (total scale), and **(d)** pCO_2_ (µatm) during the mesocosm experiment. The vertical grey line marks the day of ocean alkalinity enhancement (OAE) application (Day 6) and subsequent nutrient enrichment (Day 25).

**Fig S3.** Temporal variations of dissolved nutrient concentrations during the mesocosm experiment: **(a)** silicate (Si(OH)_4_; µmol L^-1^), **(b)** nitrate (NO_3_^-^; µmol L^-1^), **(c)** nitrite (NO_2_^-^; µmol L^-1^), and **(d)** phosphate (PO_4_^3-^; µmol L^-1^) in Ca- and Si-based OAE treatments across ΔTA levels. The vertical grey line marks the day of ocean alkalinity enhancement (OAE) application (Day 6) and subsequent nutrient enrichment (Day 25).

**Fig S4.** Larvacean (Oikopleura dioica) abundances in small **(a)** and intermediate **(b)** size classes as a function of total alkalinity increase (ΔTA) during each experimental phase. Data are shown as averages for Phase I (Days 7-25), Phase II (Days 26-53), and the combined period.

**Fig S5.** Temporal variation in **(a)** chlorophyll a concentration, **(b)** picoplankton abundance, and **(c)** predatory zooplankton abundance, including copepods, chaetognaths, and hydromedusae.

**Fig S6.** Relationships between larvacean abundance (m^-3^) and sediment-trap export material (dry weight, 48h^-1^) expressed as log_10_ (x+1) for *Phase I*, *Phase II*, and the total experimental period based on Pearson´s correlation analysis.

We investigated whether larvacean abundance was related to vertical particle export by analysing sediment trap data. During the first phase of the experiment (Days 7-25), we found a statistically significant positive correlation between export flux and larvacean abundance (r = 0.26, *p* = 0.001) based on log_10_ (x+1) transformed data. This suggests that higher dry mass collected by the traps was associated with a greater abundance of larvaceans. In Phase II (Days 26-53), this relationship weakened and was no longer significant (r = 0.08, *p* = 0.61). It is noted that during phase II, total dry matter export was much lower, which might have masked some of the effects observed during phase I. However, combining all sampling days (total period), the positive relationship re-emerged (r = 0.26, *p* = 0.02). These results suggest that larvaceans enhance carbon export. In contrast, in Phase II, although larvacean abundance was on average higher, there was no clear relationship with exported dry matter. This suggests that other sources contributed to particulate carbon flux during that period, and or grazer communities consumed houses and masked effects.

**Table S1.** Type III ANCOVA results for *Oikopleura dioica* abundance (response variable: log_10_(total abundance + 1); see Fig. 1b) in relation to ocean alkalinity enhancement (OAE). ΔTA (µmol kg^-1^) was treated as a continuous predictor, and mineral type (Ca-based vs. Si-based) as a categorical factor. MS, mean square; df, degrees of freedom; F, F statistic; *p*, p value. Significance threshold α = 0.05.

| **Experimental phase** | **Source of variation** | **MS** | **df** | **F** | ***p*** |
| --- | --- | --- | --- | --- | --- |
| *Phase I* | ΔTA | 0.014 | 1 | 0.80 | 0.403 |
|  | Mineral | 0.055 | 1 | 3.28 | 0.120 |
|  | ΔTA × Mineral | 0.073 | 1 | 4.37 | 0.082 |
| *Phase II* | ΔTA | 0.001 | 1 | 0.02 | 0.888 |
|  | Mineral | 0.003 | 1 | 0.08 | 0.789 |
|  | ΔTA × Mineral | 0.001 | 1 | 0.03 | 0.877 |
| *Total period* | ΔTA | 0.004 | 1 | 0.69 | 0.438 |
|  | Mineral | 0.000 | 1 | 0.01 | 0.913 |
|  | ΔTA × Mineral | 0.009 | 1 | 1.50 | 0.267 |

**Table S2.** Type III ANCOVA results for the contribution of *Oikopleura dioica* to total zooplankton abundance (%) under OAE (see Fig. 2b). ΔTA was included as a continuous covariate and Mineral as a fixed factor. MS, mean square; df, degrees of freedom; F, F statistic; *p*, p value. Significance threshold α = 0.05.

| **Experimental phase** | **Source of variation** | **MS** | **df** | **F** | ***p*** |
| --- | --- | --- | --- | --- | --- |
| *Phase I* | ΔTA | 0.006 | 1 | 1.07 | 0.342 |
|  | Mineral | 0.057 | 1 | 9.61 | 0.021 |
|  | ΔTA × Mineral | 0.054 | 1 | 9.01 | 0.024 |
| *Phase II* | ΔTA | 0.000 | 1 | 0.003 | 0.959 |
|  | Mineral | 0.000 | 1 | 0.002 | 0.970 |
|  | ΔTA × Mineral | 0.002 | 1 | 0.07 | 0.806 |
| *Total period* | ΔTA | 0.000 | 1 | 0.001 | 0.976 |
|  | Mineral | 0.003 | 1 | 0.23 | 0.648 |
|  | ΔTA × Mineral | 0.007 | 1 | 0.59 | 0.473 |

**Table S3.** Type III ANCOVA examining the effects of total alkalinity increase (ΔTA, continuous) and alkalinity source mineral (categorical) on three larvacean size classes (small, intermediate, large). Results correspond to Figure 3b and Supplementary Figures S1a-b. MS, mean square; df, degrees of freedom; F, F statistic; *p*, p value. Significance threshold α = 0.05.

| Larvacean size class | Experimental phase | Source of variation | MS | df | F | *p* |
| --- | --- | --- | --- | --- | --- | --- |
| Small | *Phase I* | ΔTA | 0.032 | 1 | 0.66 | 0.449 |
|  |  | Mineral | 0.000 | 1 | 0.003 | 0.959 |
|  |  | ΔTA × Mineral | 0.001 | 1 | 0.01 | 0.909 |
|  | *Phase* *II* | ΔTA | 0.071 | 1 | 1.63 | 0,248 |
|  |  | Mineral | 0.006 | 1 | 0.15 | 0.717 |
|  |  | ΔTA × Mineral | 0.008 | 1 | 0.19 | 0.682 |
|  | *Total period* | ΔTA | 0.022 | 1 | 1.55 | 0.260 |
|  |  | Mineral | 0.003 | 1 | 0.18 | 0.687 |
|  |  | ΔTA × Mineral | 0.000 | 1 | 0.02 | 0.884 |
| Intermediate | *Phase I* | ΔTA | 0.003 | 1 | 0.06 | 0.818 |
|  |  | Mineral | 0.014 | 1 | 0.28 | 0.616 |
|  |  | ΔTA × Mineral | 0.003 | 1 | 0.05 | 0.831 |
|  | *Phase II* | ΔTA | 0.028 | 1 | 0.88 | 0.384 |
|  |  | Mineral | 0.097 | 1 | 3.08 | 0.130 |
|  |  | ΔTA × Mineral | 0.104 | 1 | 3.30 | 0.119 |
|  | *Total period* | ΔTA | 0.002 | 1 | 0.19 | 0.682 |
|  |  | Mineral | 0.001 | 1 | 0.06 | 0.812 |
|  |  | ΔTA × Mineral | 0.012 | 1 | 1.27 | 0.304 |
| Large | *Phase I* | ΔTA | 0.001 | 1 | 0.02 | 0.898 |
|  |  | Mineral | 0.001 | 1 | 0.01 | 0.925 |
|  |  | ΔTA × Mineral | 0.020 | 1 | 0.30 | 0.605 |
|  | *Phase II* | ΔTA | 0.077 | 1 | 1.84 | 0.224 |
|  |  | **Mineral** | **0.289** | **1** | **6.90** | **0.039** |
|  |  | **ΔTA × Mineral** | **0.251** | **1** | **5.10** | **0.050** |
|  | *Total period* | ΔTA | 0.002 | 1 | 0.18 | 0.687 |
|  |  | Mineral | 0.017 | 1 | 1.74 | 0.235 |
|  |  | ΔTA × Mineral | 0.044 | 1 | 4.58 | 0.076 |

**Table S4.** Type III ANCOVA examining the effects of total alkalinity increase (ΔTA, continuous) and alkalinity source mineral (categorical) on the house production and clearance rates of *Oikopleura dioica* under OAE. Results correspond to Figure 4a-b. MS, mean square; df, degrees of freedom; F, F statistic; *p*, p value. Significance threshold α = 0.05.

| **Response variable** | **Source of variation** | **MS** | **df** | **F** | ***P*** |
| --- | --- | --- | --- | --- | --- |
| House production rate | ΔTA | 0.964 | 1 | 4.11 | 0.089 |
|  | Mineral | 0.184 | 1 | 0.79 | 0.409 |
|  | ΔTA × Mineral | 1.109 | 1 | 4.73 | 0.072 |
| Clearance rate | ΔTA | 0.021 | 1 | 0.78 | 0.410 |
|  | Mineral | 0.002 | 1 | 0.08 | 0.781 |
|  | ΔTA × Mineral | 0.003 | 1 | 0.13 | 0.730 |

**Table S5.** Type III ANCOVA examining the effects of total alkalinity increase (ΔTA, continuous) and alkalinity source mineral (categorical) on prey availability and predatory zooplankton abundance of larvacean under OAE. Results correspond to Figure 5a-c. MS, mean square; df, degrees of freedom; F, F statistic; *p*, p value. Significance threshold α = 0.05.

| **Response variable** | **Source of variation** | **MS** | **df** | **F** | ***P*** |
| --- | --- | --- | --- | --- | --- |
| Chlorophyll *a* concentration | ΔTA | 0.003 | 1 | 0.28 | 0.613 |
|  | Mineral | 0.012 | 1 | 1.01 | 0.353 |
|  | ΔTA × Mineral | 0.009 | 1 | 0.73 | 0.426 |
| Picoplankton abundance | ΔTA | 0.00 | 1 | 0.00 | 0.951 |
|  | **Mineral** | **0.022** | **1** | **6.54** | **0.043** |
|  | ΔTA × Mineral | 0.0001 | 1 | 0.02 | 0.886 |
| Predatory zooplankton abundance | ΔTA | 24073 | 1 | 0.71 | 0.432 |
|  | Mineral | 37794 | 1 | 1.11 | 0.332 |
|  | ΔTA × Mineral | 65438 | 1 | 1.93 | 0.214 |
